# Two-Photon Polarization Dependent Spectroscopy in Chirality: A Novel Experimental-Theoretical Approach to Study Optically Active Systems

**DOI:** 10.3390/molecules16043315

**Published:** 2011-04-18

**Authors:** Florencio E. Hernández, Antonio Rizzo

**Affiliations:** 1 Department of Chemistry, University of Central Florida, P. O. Box 162366, Orlando, FL 382616, USA; 2 The College of Optics and Photonics, CREOL University of Central Florida, P. O. Box 162366, Orlando, FL 382616, USA; 3 Consiglio Nazionale delle Ricerche (CNR), Istituto per i Processi Chimico Fisici (IPCF-CNR), UoS di Pisa, Area della Ricerca, Via G. Moruzzi 1, I-56124 Pisa, Italy

**Keywords:** Non-linear spectroscopy, circular dichroism, two-photon absorption, chirality, bi-naphthols

## Abstract

Many phenomena, including life itself and its biochemical foundations are fundamentally rooted in chirality. Combinatorial methodologies for catalyst discovery and optimization remain an invaluable tool for gaining access to enantiomerically pure compounds in the development of pharmaceuticals, agrochemicals, and flavors. Some exotic metamaterials exhibiting negative refractive index at optical frequencies are based on chiral structures. Chiroptical activity is commonly quantified in terms of circular dichroism (CD) and optical rotatory dispersion (ORD). However, the linear nature of these effects limits their application in the far and near-UV region in highly absorbing and scattering biological systems. In order to surmount this barrier, in recent years we made important advancements on a novel non linear, low-scatter, long-wavelength CD approach called two-photon absorption circular dichroism (TPACD). Herein we present a descriptive analysis of the optics principles behind the experimental measurement of TPACD, *i.e.,* the double L-scan technique, and its significance using pulsed lasers. We also make an instructive examination and discuss the reliability of our theoretical-computational approach, which uses modern analytical response theory, within a Time-Dependent Density Functional Theory (TD-DFT) approach. In order to illustrate the potential of this novel spectroscopic tool, we first present the experimental and theoretical results obtained in C_2_-symmetric, axially chiral *R*-(+)-1,1'-bi(2-naphthol), *R*-BINOL, a molecule studied at the beginning of our investigation in this field. Next, we reveal some preliminary results obtained for (*R*)-3,3′-diphenyl-2,2′-bi-1-naphthol, *R*-VANOL, and (*R*)-2,2′-diphenyl-3,3′-(4-biphenanthrol), *R*-VAPOL. This family of optically active compounds has been proven to be a suitable model for the structure-property relationship study of TPACD, because its members are highly conjugated yet photo-stable, and easily derivatized at the 5- and 6-positions. With the publication of these outcomes we hope to motivate more members of the scientist community to engage in state-of-the-art TPACD spectroscopy.

## 1. Introduction

Since the very first observation of the rotatory power of tartaric acid done by Biot in 1815 [1,2,3,4], countless advances have been made in the field of molecular dissymmetry. In this particular case we are referring to chirality. The most significant progress in the understanding of chirality and its applications has occurred during the last twenty years with the establishment of circular dichroism (CD) and optical rotator dispersion (ORD). Since then, electronic circular dichroism (ECD) has mainly contributed to the study of the physical-chemical and conformational properties of optically active biomolecules [5,6]. Nonetheless, because this method is based on the one-photon absorption (OPA) of optically active compounds, which typically takes place in the far and near UV region of the electromagnetic spectrum, the OPA of standard aqueous buffer systems in the same spectral region can cover the small ECD signal, and the scattering exhibited at short wavelengths becomes an obstacle in inhomogeneous samples. Consequently, further investigation of attractive biological systems such as natural amino acids structures and proteins, and the superior engineering of unique functionalities for practical asymmetric catalysis based on chiral systems has become a challenge.

In order to overcome the existent limitations, scientists in this field have also proposed vibrational circular dichroism (VCD) [7,8,9] and Raman optical activity (ROA) [10,11,12]. So far, the IR spectroscopic approach has yielded major advances in the determination of the absolute configuration of small molecules and in the elucidation of secondary structure in proteins [13,14]. However, the analysis of many attractive biological systems such as those mentioned before [14,15], and the study of innovative optically active drugs soluble in organic solvents demand alternative approaches that can reveal unique spectroscopic and structural features at shorter wavelengths.

During the last decade, nonlinear optics have opened a new path in this direction. Processes such as sum-frequency generation (SFG) [16], second harmonic generation (SHG) [17,18], and nonlinear optical activity (NOA) [19,20], including multiphoton optical rotation [21,22,23,24,25], have been reported, experimentally and theoretically, for the study of chemical and biological chiral systems. Although, the development of these novel nonlinear optical approaches has been an important piece to solving the puzzle, truly polarization dependent multiphoton absorption processes (two-photon absorption) were experimentally unrevealed until our recent development of the double L-scan technique [26]. It is the aim of this article to present a detailed analysis of the principles behind the double L-scan technique [26] and the origin of its high sensitivity to measure two-photon absorption circular dichroism (TPACD; note that in previous papers we have used the alternative acronyms 2PA-CD and TPCD.

**Figure 1:**
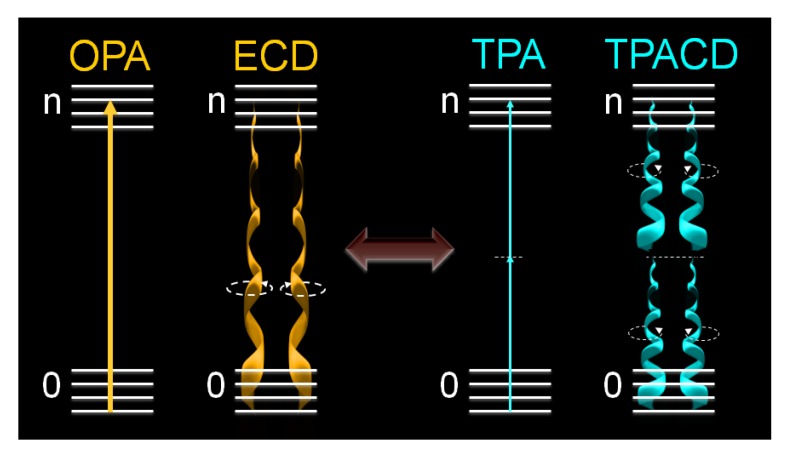
Comparative schematic between OPA and TPA processes as well as ECD and degenerate TPACD.

TPACD in optically active molecules was first proposed by Tinoco [27] and Power [28] in the 1970s. Because of its potential applications in the fundamental study of chiral systems, TPACD has regained the attention of the scientific community during the last few years [29,30,31,32,33,34,35,36]. Two-photon absorption is based on the simultaneous absorption of two photons with longer wavelength than that needed for OPA and whose sum of energies corresponds to an electronic transition [37]. In the degenerate case (two photons of equal circular frequency *ω* and associated wavelength *λ* = 2 *πc*_0 _/*ω*, with *c*_0_ standing for the speed of light *in vacuo*), TPACD is defined as 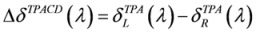, where *δ_L_^TPA^*(*λ*) and *δ_R_^TPA^*(*λ*) are the TPA cross-sections, *δ^TPA^*(*λ*), for left and right circularly polarized light, respectively (Figure 1). Because at typical TPA excitation wavelengths the linear absorption is negligible and scattering minimized, the study of short wavelengths absorbing molecules becomes advantageous [38,39]. In addition, the fact that TPA transitions obey different selection rules than OPA (even-parity *vs.* odd-parity) leads to think that in chiral molecules ECD and TPACD should present different spectral features. Of course, these rules are very strict only in centrosymmetric molecules. As a result, one should expect greater differences in optically active compounds with a center of symmetry, which is contradictory. Nevertheless, it has already been predicted theoretically that in molecules that do not satisfy this condition, such as chiral molecules, significant differences between the ECD and TPACD spectra can be observed [31]. Therefore, being able to measure TPACD in chiral compounds whose excitation energies are in the UV, would provide structural and conformational information that is complementary to that obtained using ECD. 

Hitherto, measuring TPACD in chiral molecules has represented a major challenge to scientists in the field, mainly because the dichroism is determined by the small contribution of the transition magnetic-dipole and electric-quadrupole transition moments [27,28]. Experiments using pump-probe, [19], intensity dependent multiphoton optical rotation [25], resonance-enhanced multiphoton ionization [40,41] and polarization modulation single beam Z-scan [42] have been attempted. Although all these experimental approaches were clever, none of them has been capable to fully reveal pure TPACD, and even more important, to produce a definite TPACD spectrum of any substance.

In 2008 we came up with a novel experimental approach called the double L-scan [26]. This method was inspired by the most used experimental technique employed now-a-days to measure TPA, *i.e.*, Z-scan [43]. Through the development of this highly sensitive technique, we were able to measure, for the very first time, the full TPACD spectra of bi-naphthol derivatives. The whole secret behind our unique technique, as explained below, is the simultaneous measurement of the TPA using “identical twin” pulses with different circular polarization state.

In the following we will describe in detail the experimental approach employed in our group to measure TPACD. Next, we review the theory of this novel spectroscopy, and show how, with the establishment of a computational protocol, it is possible nowadays to obtain reliable *ab initio* simulations of the TPACD spectra, also on chiral systems for which experimental data are now available. After a brief analysis of the dependence of the observable on the approximations adopted in our simulations, and a sort excursus on the computational results published in our group since the first paper in 2005 [29], we briefly review the degree of agreement between theory and experiment for BINOL, VANOL and VAPOL.

## 2. Experimental Approach for TPACD

Our first attempts to measure TPACD were completed using the classical single beam Z-scan technique [43]. Using this method we scanned the sample across the focal plane on a focusing geometry along the Z-axis, and pumped in separated runs with linearly polarized light (LPL), right circularly polarized light (RCPL), and left circularly polarized light (LCPL). For this study we chose a well know molecule with axial chirality, *i.e.*, (*S*)-(-)-1,1’-bi(2-naphthol) and (*R*)-(+)-1,1’-bi(2-naphthol) (enantiomeric ratio *R*:*S* ≥ 99:1) in THF solution, hereafter *S*-BINOL and *R*-BINOL, at a concentration of 2 × 10^−3^M. The excitation beam was generated by an optical parametric generator (OPG) pumped by the third harmonic of a mode-locked Nd:YAG laser (EKSPLA System) operating at a 10 Hz repetition rate and a pulse duration of 25 ps (FWHM). The OPG energy fluctuation was < 30% and the excitation beam had an M^2 ^≈ 1.2.

**Figure 2:**
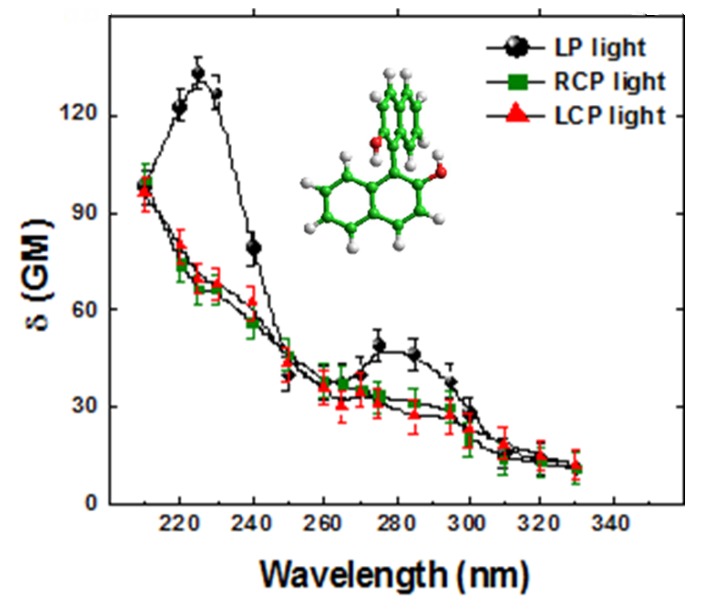
Single beam Z-scan TPA spectra of *R*-BINOL/THF pumping with RCPL (green solid squares), LCPL (red solid triangles) and LPL (black solid circles) and display at half of the excitation wavelength.

In Figure 2 we show our preliminary results. Since our OPG system was not very stable, we selected, using a homemade acquisition system, only pulses within a very narrow energy window (± 2%) and averaged hundreds of points at each position. Under these extreme experimental conditions, we were expecting to observe the anticipated small spectral differences between *δ_L_^TPA^*(*λ*) and *δ_R_^TPA^*(*λ*). However, the two signals were virtually identical within the error. In fact, we noticed that the error was greater than just 2%.

**Figure 3:**
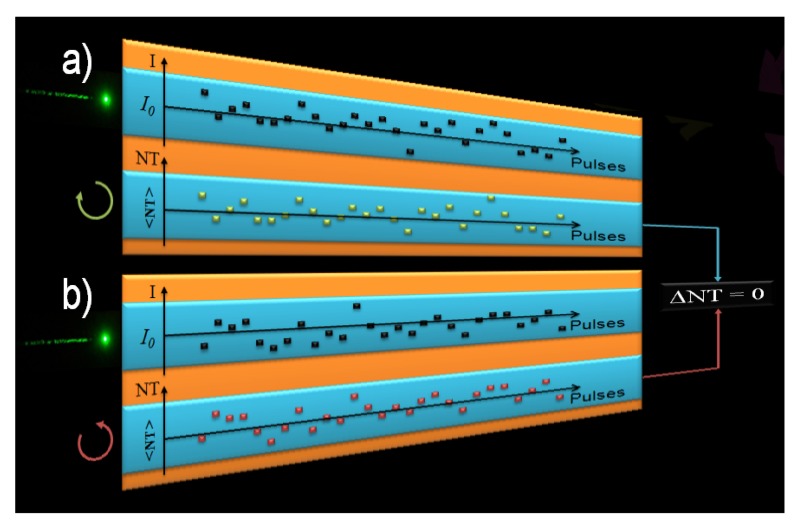
Representation of TPA measurements performed with the single beam Z-scan technique. (a) corresponds to measurements performed using RCPL and (b) LCPL. Top drawing represents the intensity (I) of the individual pulses exciting the sample and the bottom drawing represents the normalized transmittance (NT) measured trend for each individual pulse.

The results displayed in Figure 2 led us to propose the following hypothesis: significant and not uncommon beam size and shape changes, or even modal fluctuations between pulses that can affect its spatial energy distribution could change the effective irradiance at each Z position, thus inducing a bigger fluctuation of the signal. This can, therefore, mask the TPACD signal. In order to illustrate better this point, we show in Figure 3 a schematic representing the way we pictured the problem throughout the measurements of the TPA cross-sections using the single beam Z-scan [43]. We ran independent scans for different polarization states of the pump. In order to minimize the overall signal fluctuation we averaged hundreds of points maintaining constant the average energy of the laser (indicated here as the incident irradiance *I_0_*). Also, we set a narrow energy window (width of the blue box) on the acquisition system to select only pulses with energy within that range. Although these parameters remain constant in time between different runs, we could expect to measure different values of the normalized transmittance (NT) as a consequence of fluctuations in the effective intensity of the pulse. Therefore, while in one run pulse *n* might have a higher irradiance than the average (*I_0_*), due to any or a combination of the effects cited above, in another run pulse *n’* might have a lower value, and *vice-versa *[see Figure 3a) and 3b)]. Consequently, over time, if the anticipated differences are within the average fluctuations, the resultant mean-value of the normalized transmittance (<NT>) becomes virtually identical for both polarizations, thus yielding ΔNT ≈ 0. This artifact can then conceal the TPACD signal. These kinds of effects are in general overlooked in Z-scan experiments because usually the nonlinearity is greater than just few percents *δ^TPA^*(*λ*).

**Figure 4:**
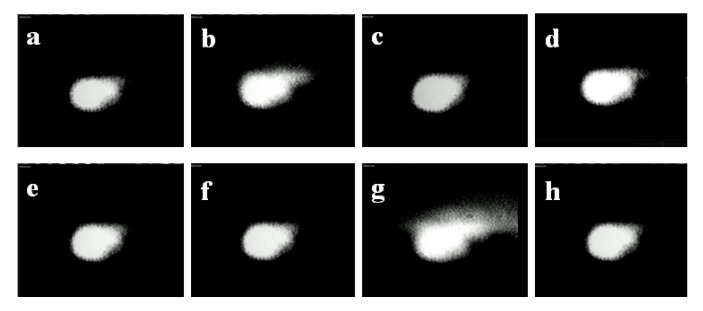
Snapshots of eight different consecutive pulses obtained with a single beam geometry.

To prove our hypothesis we measured, using a CCD camera, the beam profile for many pulses. In Figure 4, we show the snapshot of eight consecutive pulses using the single beam geometry. It is clear how the beam energy distribution, as well as its spatial profile and size, indeed changes significantly between pulses.

In order to avoid this artifact, we put forward a novel methodology to measure simultaneously RCPL and LCPL on a 90° double arm setup. This perpendicular geometry required the sample to be held fixed in one position instead of being moved across the focal plane along the *Z*-axis. Therefore, we followed a different approach, *i.e.,* to move the focuses of the two beams across the sample (S) by translating the lenses. This is why our technique is called the double L-scan. 

**Figure 5:**
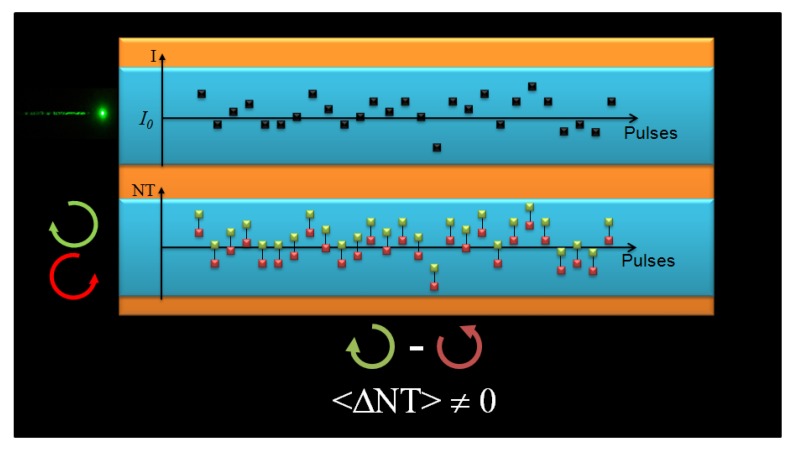
Depiction of TPA measurements performed using the double L-scan technique. Excitation using RCPL (green solid squares) and LCPL (red solid squares). Top represents the intensity (I) of the individual pulses exciting the sample and bottom represents the normalized transmittance (NT) measured trend for each individual pulse for both circular polarizations.

In Figure 5, we show an expounding picture of the basic principles of the double L-scan. We performed this experiment using two identical (twins) beams generated by the same seed pulse. These beams travel identical paths and traverse virtually the same optical elements. This guarantees that the beam spatial fluctuations at the sample are identical through both arms. Therefore, when pulse *n* hits the sample with a higher irradiance than the average preselected value (*I_0_*), due for instance to beam spatial redistribution of the energy, both signals will follow the same tendency, thus given a stronger signal. The same applies the other way around, *i.e.*, when another pulse *n’* hits the samples with a lower irradiance than the average preselected value *I_0_*, the expected signal for both beams will be lower. As a result and to our advantage, the difference between the two signals will always be present (ΔNT ≠ 0) and shall retain the sign. Therefore, TPACD can only be obtained by averaging over ΔNTs of the individual pulses.

**Figure 6:**
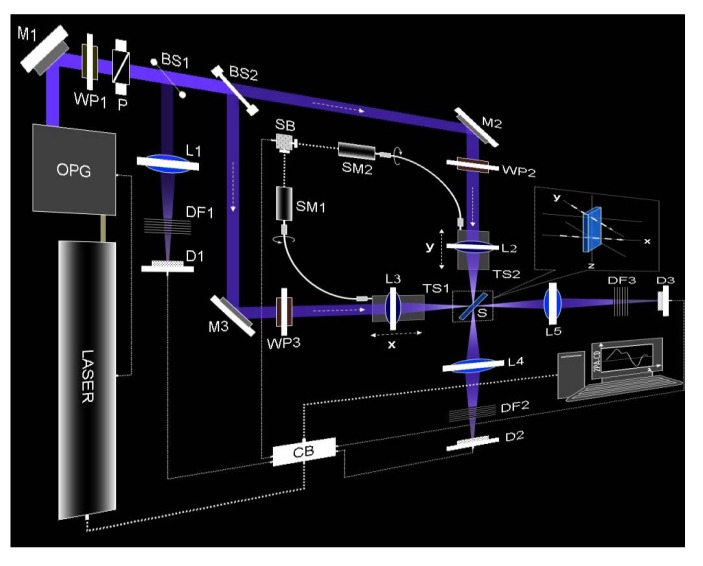
Experimental setup of the double L-scan. Between the laser system and mirror M1 more optical elements such as converging and diverging lenses, pinholes, optical filters, polarizers and waveplates were placed for beam expansion, collimation, spatial filtering, energy attenuation and polarization control. The power of the pump beam is attenuated and linearly polarized using a combination of an achromatic zero-order broadband (spectral range ≈ 400–700 nm) half-wave plate (WP1) and a broadband Glan polarizer (P). The beam splitter BS1 (10/90) is employed to constantly monitor a weak portion of the incoming laser light as an energy reference on a silicon detector (D1). The strongest fraction of the laser is separated into two identical beams using a broadband (50/50) beam splitter (BS2). The final alignment of the two beams is controlled by means of two broadband aluminum mirrors (M2 and M3). The polarization state of the incident beams after all reflective elements, is achieved by placing two achromatic zero-order broadband quarter-waveplates (400–700 nm), WP2 and WP3, before two achromatic convergent lenses, L2 and L3 (focal distance = 100 mm, w0 ≈ 11 μm). L2 and L3 are moved simultaneously, with identical displacement x = y, and in the same direction on two individual translation stages (TS1 and TS2). TS1 and TS2 are simultaneously translated by coordinating two step-motors, SM1 and SM2, using a synchronization box (SB). Due to the experimental geometry, S is placed at a 45° angle with respect to the incident beams propagation axes, giving an effective path length equal to 1.4 mm (1 mm thickness cell). The two incident beams are approximately 1 cm apart on the vertical axis (inset) and both are parallel to the table. After the sample, L4 and L5 are utilized to fully collect the total energy of the beams into silicon detectors D2 and D3. Neutral density filters (DF1, DF2, and DF3) are used to adjust the input energy into the different detectors. The whole setup is synchronized and controlled with a control box (CB) and a LABVIEW program using a PC [26].

In Figure 6 we show the detailed diagram of the double L-scan setup reported in reference [26]. For this part of the experiment we used the same EKSPLA system described before and did measurements on a similar solution of R-BINOL/THF. One should notice that the two arms of the double L-scan are a perfect replica of each other.

**Figure 7:**
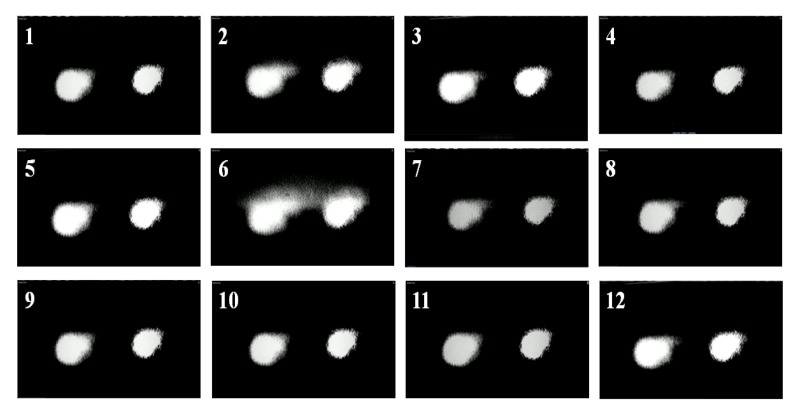
Snapshots of twelve different consecutive pulse pairs measured through a double L-scan geometry. Differences in size between pulses in the same picture are due the angle at which the CCD was placed during the measurements.

With the double L-scan in place we then measured, simultaneously, changes on the two beams profile and energy distribution for many pulses (see video in supporting information). The CCD was placed at approximately 45° with respect to both beams, close to the sample position. In Figure 7, we show the snapshot of twelve consecutive pair of pulses using this novel geometry. The small difference in size between the pulse on the left and right side of the pictures are just due to camera position and angle, and not due to the experimental dissimilarities. From this figure it is clear that energy redistribution as well as spatial profile and size changes are indeed identical for both beams. Hence, our geometry accounts for changes in laser performance by splitting the original pulse into mirror image pulses, which are subject to the same type of spatial and temporal fluctuations of the pulsed source. This approach alleviates the concerns associated with changes in the pulse profile between runs when performing TPACD.

Since there is a need to set exactly the same experimental conditions through both arms before measuring the TPA cross section values on any sample, calibration of the double L-scan is required. The procedure to certify the achievements of the desired indistinguishable settings consists of measuring the TPA of the sample under study, using linear polarization in both arms. This is the obvious case for which ΔNT would be equal to zero. Having found this condition one can proceed to perform measurements for different combinations of polarization states (RCPL and LCPL). These states are achieved by only rotating the extraordinary axis of WP2 and WP3 with respect to the plane of polarization (vertical) of the incident beam.

**Figure 8:**
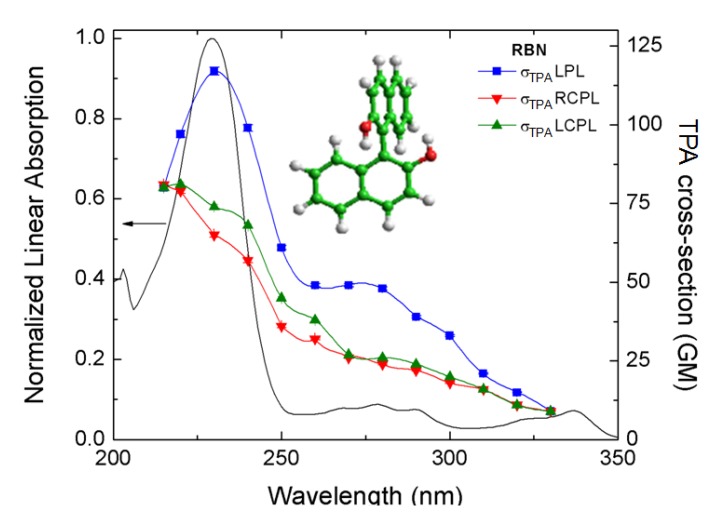
Double L-scan TPA spectra of *R*-BINOL/THF pumping with RCPL (red solid triangles), LCPL (green solid triangles) and LPL (blue solid squares) and display at half of the excitation wavelength. The normalized linear spectrum is also plotted for comparison (black solid line) [44].

Figure 8, shows the TPA spectra of *R*-BINOL/THF measured with the double L-scan using different polarization states [44]. The experimental error bars are within the size of the symbols (<1%). All TPA data are plotted at half of their excitation wavelength for direct comparison with the linear absorption spectrum. The first evident observation is the big difference in magnitude between the TPA cross sections measured with LPL and RCPL and/or LCPL. This difference persists over most of the spectral range analyzed. However, the most important point to underline from these results is the quantifiable difference between *δ_L_^TPA^*(*λ*) and *δ_R_^TPA^*(*λ*).

**Figure 9:**
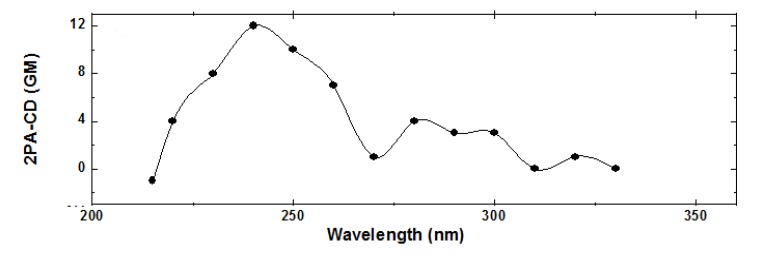
TPACD spectrum of *R*-BINOL/THF.

From the data displayed in Figure 8 we calculated the TPACD spectrum of *R*-BINOL (see Figure 9). On the one hand, it is noticeable the TPACD spectrum positive sign down to approximately 216 nm. On the other hand, at shorter wavelengths it changes its sign to negative. The origin of these and other spectral features have already been analyzed in Ref. [44]. Notable to mention is the agreement obtained between the experimental and theoretical spectra reported in the same article.

**Figure 10:**
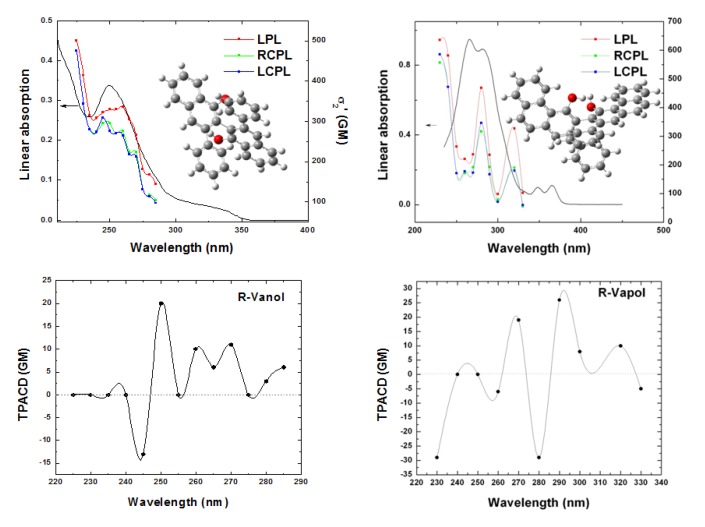
OPA and TPA spectra of *R*-VANOL/THF (top left) and *R*-VAPOL/THF (top right), exciting with different polarization states, LPL (red solid squares), RCPL (green solid squares) and LCPL (blue solid squares). TPACD spectra of *R*-VANOL/THF (bottom left) and *R*-VAPOL/THF (bottom right).

To end this section, in Figure 10 we show the measured OPA, TPA and TPACD spectra obtained for the *R*- enantiomers of 3,3′-diphenyl-2,2′-bi-1-naphthol (VANOL), and 2,2′-diphenyl-3,3′-(4-biphenanthrol) (VANOL). These biaryls, closely related to BINOL, have been recently the subject of a combined experimental-theoretical study presented in Ref. [45]. Some of those results will be presented at the end of this work.

## 3. Theory of TPACD

The expression for TPACD, defined as 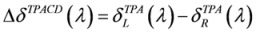, was obtained by Tinoco in his 1975 paper as a semiclassical extension of the TPA formulae [27]. Quantum electrodynamical equivalent expressions were obtained by Power [28], by Andrews [46] and, in a series of papers, by Meath and Power [47,48,49,50], who were able to generalize the approach to the case of *n* photons [49], and considered also the modifications occurring in the formulae when elliptical polarization is assumed [50]. A nice discussion of the theoretical foundation of TPACD can be found in the book of Lin and co-workers [51]. In 1986 Szłucki and Stręk [52] predicted the possibility of detection of TPACD in the fluorescence of lanthanides (observed in 1995 by Gunde and Richardson [53]).

When working within the pure electric dipole approximation, the polarization dependent two-photon absorption strength has the same expression for left and right circularly photons. Therefore, as for the linear electronic circular dichroism [54,55,56,57], it is only when at least magnetic dipole and electric quadrupole interactions are taken into account that a non vanishing dichroism, Δ*δ^TPCDA^*(*λ*), can be predicted, and then only for chiral structures. Following the original expression obtained by Tinoco [27], for transitions from the ground electronic state |0〉, involving the excited electronic states |*n*〉, employing a velocity operator formulation, and therefore yielding origin invariant results in approximate calculations, the TPACD is usually expressed as:

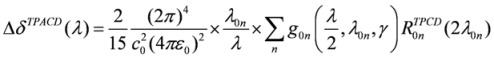
(1)

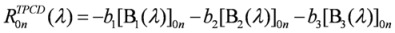
(2)
where *ε*_0_ the electric constant and 
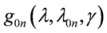
 is an appropriate lineshape function, depending on the wavelength *λ*, the transition wavelength 
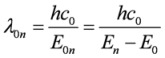
 and the parameter *γ* discussed just below (*E*_0*n*_ denotes the excitation energy). In practice, in our calculations the lineshape function used is either a Lorentzian:

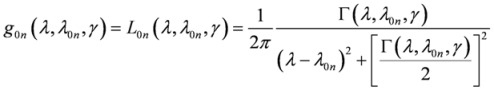
(3)
or a Gaussian:

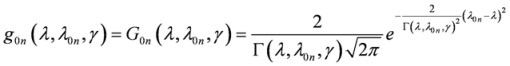
(4)
where we have:

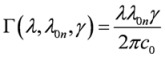
(5)


*γ* is a phenomenological parameter, whose units are those of an inverse of time, and which corresponds to the FWHM for a Lorentzian expressed as a function of the circular frequency *ω*, or to the FWHM divided by 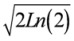 for a Gaussian again expressed as a function of the *ω *(both functions are therefore normalized to unity on the *ω* axis). With the definition of Eqs. (3) and (4) each lineshape function is not normalized to unity in the wavelength domain, and it is not symmetric with respect to the *λ* =*λ*_0*n*_ coordinate, where the function has its maximum. Quite often one approximates 
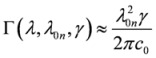
, which restores both symmetry and normalization in the wavelength domain. 

Going back to Eqs. (1) and (2), the parameters *b*_1_, *b*_2_ and *b*_3_ are characteristic of a given combination of polarization and mutual direction choices for the two absorbed photons, one of which needs to be left or right polarized. B_1_(*λ*), B_2_(*λ*) and B_3_(*λ*) can be written as follows:

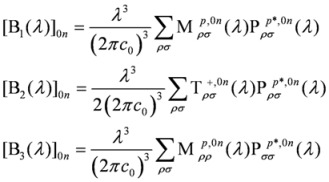
(6)


and they involve generalized two-photon transition tensors, which, for two photons of the same frequency, are defined as:

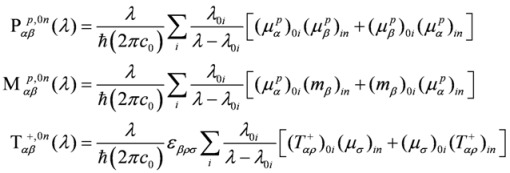
(7)


Here *ε*_αβγ _denotes denotes the Levi-Civita alternating tensor, and the notation 
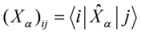
 indicates the matrix element between electronic states |*i*〉and |*j*〉of the *α*-component of the operator 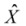, which is either the velocity operator 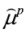:

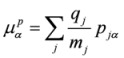
(8)
the magnetic dipole operator 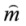:

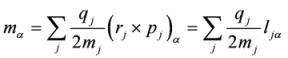
(9)


and the velocity form of the electric quadrupole operator 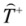:

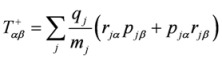
(10)


Summations in the last formulae run in principle over all particles of mass *m_j_*, position *r_j_*, linear momentum *p_j_* and angular momentum *l_j_*. In Ref. [30] alternative formulations were presented, including a full “length” approach which involves transition matrix elements of the “more usual” electric dipole and electric quadrupole operators, but bearing the disadvantage of yielding results that depend upon the choice of the origin of the multipolar expansion. 

Note that, contrary to what happens for the linear electronic circular dichroism, the quadrupolar interaction does play a role in TPACD even for isotropic samples, albeit to date no examples are known of systems where this role appears to be really relevant. It can be easily shown that the ratio in intensities between TPACD and corresponding TPA signals is on the order of 
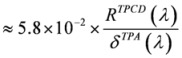
, and both the rotatory and the transition strengths are assumed to be given in a.u. (where they are usually of comparable size).

## 4. The Computational Approach to TPACD

Exactly as the theoretical expressions of the dichroism observed in the absorption of two photons could be obtained as an extension of the formulae yielding the two-photon absorption rate [27], also the computational protocol allowing for the calculation of TPACD profiles was derived by a relatively simple extension of the approaches commonly employed for calculations of two-photon absorption spectra. In particular, modern analytical response theory [58,59,60] has given for the last twenty five years a very efficient computational tool for the direct calculation of the two-photon transition amplitudes, as the single residue of the appropriate quadratic response function [59,60]. Indeed, in Ref. [29] it was shown that the molecular parameters entering the TPACD rotatory strengths Eq. (2), given in Eqs. (7), can be obtained, as for the two-photon transition amplitude, as single residues of appropriate quadratic response functions:

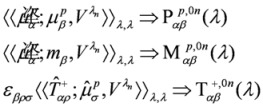
(11)
where *V*^λ*n*^ is an arbitrary perturbation associated with the excitation vector representing excited state |*n*〉. A generalization of the routines originally written to compute the standard two-photon tensor within the DALTON suite [61] allowed the authors of Ref. [29] to obtain simulated TPACD spectra for the isolated molecules of (L)-tryptophan and (L)-tyrosine. An origin invariant estimate of the dichroism observed in a two-photon absorption process on a chiral assembly involves therefore the calculation of three instead of one single residues when compared to the corresponding TPA. If the appropriate response equations are solved, and the residues computed all in the same run, that is without recomputing the ground state wave function, TPACD can be obtained, *together* with TPA, at roughly four times the cost of standard TPA. To date, we are not aware of alternative routes to TPACD besides the one open by one of the present authors, with his collaborators, and exploiting the capabilities of the DALTON suite [29].

As an immediate consequence of the approach followed when implementing in DALTON all the additional drivers needed, TPACD has been in principle available since its (computational) beginnings for the whole list of electronic structure models available within DALTON, ranging from HF-SCF [58,62], to MCSCF [62], the whole hierarchy of Coupled Cluster methods [63,64] and DFT [65]. To date, only TDHF (Random Phase Approximation, RPA) and TD-DFT have been employed [29,31,32,34,36,66], as the size and reduced symmetry of the systems exhibiting either intrinsic or structural chirality studied computationally make them unsuitable for more refined ab initio wave function models. The effect of electron correlation on the property has been therefore ascertained by comparing RPA and TD-DFT results. For the latter, only the Becke-three parameters Lee, Yang and Parr (B3LYP) [67,68,69] and Coulomb attenuated method-B3LYP (CAM-B3LYP). [70,71,72] Exchange Correlation functionals have been tested in the very few cases (the study on *R*-(+)-1,1'-bi(2-naphthol), *R*-BINOL, presented in Refs. [44,66] and that on (*R*)-3,3′-diphenyl-2,2′-bi-1-naphthol, *R*-VANOL, and (*R*)-2,2′-diphenyl-3,3′-(4-biphenanthrol), *R*-VAPOL, whose outcome is partly anticipated in this review, where a comparison with experiment could be made. In the case of *R*-BINOL, in particular, the computational challenges which are to be met when studying TPACD have been analyzed in depth [66].

The need to account properly for electron correlation effects, which is especially important for a nonlinear property as TPACD involving electric dipole, electric quadrupole and magnetic dipole interactions, imposes, besides the use of reliable wave function models, also that of extended basis function sets. This is of particular importance when considering that the origin invariant approach proposed by Tinoco, and conveniently employed in most cases, involves the calculation of transition matrix elements of the velocity forms of the electric dipole and quadrupole operators [30]. Correlation consistent basis function sets of double zeta quality, singly augmented, have been employed in the vast majority of the cases studied to date. This aspect has been analyzed in some detail in Ref. [30].

The ability of modeling accurately the effect of the environment when simulating TPACD spectra is of paramount importance, since practically all chiral systems, structures and aggregates of relevance in life science and in all other areas of science and technology are found in a condensed phase. Condensed phase effects have been conveniently and quite satisfactorily accounted for in TPACD studies by resorting to the Polarizable Continuum Model, PCM [73,74], also available within the DALTON suite. In the simulations of the TPACD spectra of (R)-3-Methyl-Cyclopentanone of Ref. [34] and of that of (L)-tryptophan [36] a PCM was employed to account for the different responses of the solvated species. Moreover, in those studies another important aspect that needs to be carefully taken into account, that is the distribution of conformers with similar energy and widely different response to the external perturbation, was discussed. In the study of Ref. [34] this required proper Boltzmann averaging between two dominant conformers, whereas in Ref. [36] the analysis involved both neutral and zwitterionic forms, and was extended to ten conformers in the gas phase and nine in the dielectric medium.

Another aspect of this rather new spectroscopy, analyzed theoretically and computationally, is that of vibronic effects, which can strongly influence intensities and shapes of the electronic TPACD spectra. These have also been studied recently by resorting to rather sophisticated perturbative approaches [35].

## 5. Early Computational Studies and Comparison of Experiment and Theory: BINOL, VANOL, VAPOL

In the early computational studies of TPACD attention was focused on the proteinogenic amino acids [29,31], the aim being to convince the scientific community that the new, then only “in silico” spectroscopic tool might be an attractive alternative to other techniques for biologically relevant samples. Indeed those initial accounts, besides predicting the measurability of the dichroism, could prove the fingerprinting capabilities of TPCD, yielding information often complementary to that obtained by other spectroscopies, and in particular by ECD. As mentioned in the previous section, in a more recent extremely detailed follow up, focusing on a particular amino acid – (L)-tryptophan – [36] the effects of conformational variety and that of solvation were explored, highlighting the wide range of responses across the various conformers, and the remarkable sensitivity to the often relatively minor changes in the geometry induced by the electrostatic interaction with the molecules of the solvent.

Similar conclusions were also reached in our study of (*R*)-(+)-methylcyclopentanone [34], motivated by the interest in nonlinear dichroic response of the molecule revealed by the resonance enhanced multiphoton ionization CD (REMPI-CD) studies by the group of Compton [40,75] and those on CD in Laser Mass Spectroscopy by Boesl von Grafenstein and Bornschlegl [76,41]. Nevertheless in this case the peculiar electronic structure of the molecule – with well characterized low lying excited electronic states and a relatively simple conformational manifold (both in the gas and in the solvated medium the molecule exhibits two dominant conformations with a ratio of 9:1 around room temperature) allowed for an in depth analysis, extending to the study of the vibronic effects [35]. It was shown that the effect of molecular vibrations can be in some case so strong as to yield a change of the sign of the dichroic signal assigned to a given excited electronic state. This is an effect that can be described theoretically only by going beyond the often employed Frank-Condon approximation, and introducing the so called Hertzberg-Teller couplings and possibly also Dushinski rotation effects [35].

In the quest for structures with particularly strong two-photon circular dichroism response attention has been given to systems with structural chirality, as the helicenes and, more recently, the biaryls (BINOL, VANOL, VAPOL). In the study of Ref. [32], centered around four different [M]-helicene structures, it was shown that a unique combination of chirality and electron delocalization effect can give rise to strong TPCD responses, to the point that a ratio of TPA vs. TPCD intensities between one and two orders of magnitudes was computed for the classical structure of [6]-helicene (hexahelicene).

**Figure 11:**
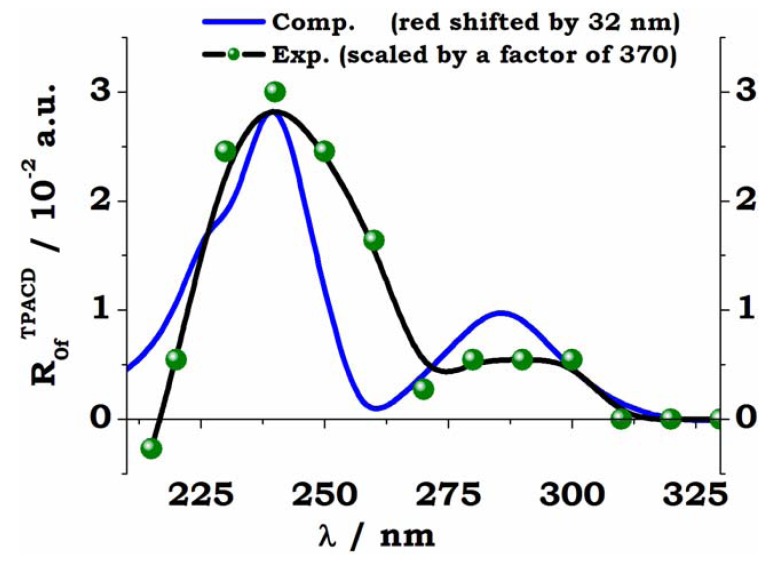
TPCD. Two circularly polarized photons. Comparison of experimental (black curve, B-spline interpolation of the experimental points) and theoretical (blue curve, PCM/DFT/CAM-B3LYP results, aug-cc-pVDZ basis set) for *R*-BINOL in THF. In the abscissa the one-photon wave length. Gaussians functions *G*(*λ*, *λ_0n_*, *γ*) with FWHM 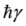 = 0.3 eV employed for the simulation, fee Ref. [66].

For R-BINOL it was possible finally to put the computational protocol to a test. The calculations involved as many as thirty excited electronic states, which permitted an analysis of the spectrum throughout the whole range explored in experiment (see Figure 9), that is as far down as 200 nm [66]. The comparison between the spectrum, simulated at TD-DFT/CAM-B3LYP level, using an aug-cc-PVDZ basis set and PCM to mimic the effect of the solvent (THF) is given in Figure 11.

The simulated curve in Figure 11 is shifted towards the red by 32 nm, in order to maximize the overlap with experiment, which, in turn, is scaled in intensity by a remarkable factor of 370. The tendency of CAM-B3LYP as a functional to overestimate the excitation energy (in particular for valence excitations) is well known [77,78]. It is also true that CAM-B3LYP was recently shown to be preferable to other functionals, and in particular to B3LYP, for TD-DFT calculations of excitation energies and properties, especially involving charge transfer (CT) states, since it can give a balanced description of local, Rydberg, and CT excitations [79]. As far as the huge scaling factor employed in the Figure, an in depth discussion of the possible causes of the discrepancies between computed and measured intensities in TPCD can be found in Ref. [66], where it was shown that these cannot be attributed to geometrical or conformational effects, or to deficiencies in accounting for excitonic or CT couplings. It is to be noted that the corresponding simulation of the linear ECD spectrum of R-BINOL nicely agrees with experiment [80].

The combination of CAM-B3LYP and PCM appears to quite satisfactorily reproduce both the separation between the two experimental maxima, the shallow plateau at *λ*≈ 90 nm and the sharp more intense peak at *λ*≈ 40 nm, and their relative intensity. For a detailed analysis of these results, and a discussion of the performance of the B3LYP the interested reader should consult Ref. [66], where the effect of the slow scissoring motion of the two naphtyl moieties is also analyzed, together with the consequences of the pairing structure of the electronic excited states arising from the week interaction between the two monomeric units.

**Figure 12:**
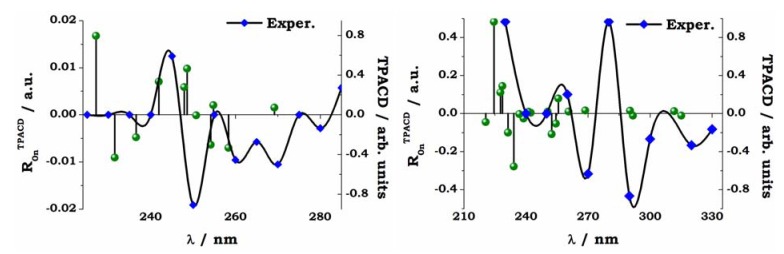
*S*-VANOL (left panel) and *S*-VAPOL (right panel). Comparison of experiment and theory for the TPCD spectra. The vertical sticks are the *ab initio* TPCD rotatory strengths (*R_0n_^TPACD^*), computed for the lowest twenty excited electronic states. The computed excitation energies are red shifted by 20 nm in S-VANOL, by 10 nm in *S*-VAPOL. These values were chosen to bring the maximum of OPA peaks as close as possible to the experimental peak located around 250 nm (*S*-VANOL) and 290 nm (*S*-VAPOL). See Ref. [45].

In close, we report some preliminary results for the comparison between theory and experiment two typical biaryls, *R*-VANOL and *R*-VAPOL, whose structures are shown, together with the experimental curves, in the inset to Figure 10. The VCD, ECD and ORD spectra of VANOL [81] and VAPOL [82] have been studied recently by Polavarapu and co-workers, who employed their spectroscopic evidences to determine the absolute configurations of these two by-aryls. Here calculations for these two molecules were carried out on the S- enantiomers with geometries optimized at B3LYP/6-31G* level, in both gas and dichloromethane (the solvent employed in experiment). For the TD-DFT calculation the same basis set (6-31G*) was employed. The size of the systems (54 nuclei, 230 electrons for VANOL, 68 nuclei, 282 electrons for VAPOL) made the use of a larger basis set rather unpractical. Spectra were simulated, again for the isolated molecules and for solutions in dichloromethane, exploiting both the B3LYP and the CAM-B3LYP functional. Here we compare experimental and simulated PCM/CAM-B3LYP spectra for TPACD, see Figure 12.

A detailed analysis of the results shown in Figure 10, and of their comparison with experiment for the whole set of OPA, ECD, TPA and TPACD results is reported elsewhere [45]. As for *R*-BINOL, both the measurement and the simulation of the non linear dichroic response of large systems as these biaryls are a formidable challenge for both experimentalists and computational chemists. Nevertheless, the analysis of the relationships between measured and computed spectra in Figure 12, where the computed stick spectrum is properly shifted in order to maximize the overlap between the most relevant peaks shown in linear absorption, see Figure 10, shows, in particular for *R*-VANOL, that the alternation of negative and positive peaks is somewhat reproduced. The strongest TPACD response appears in the blue section of the spectra, and it involves therefore higher lying excited electronic states. One state (around 230 nm, see right panel in Figure 12) dominates for *R*-VAPOL. This is a region which is not reached by our experiment and clearly at the boundary of the range of excited states involved in our TD DFT study. Also for VAPOL the simulation appears to yield an alternation of positive and negative peaks, although the relative intensity of the peaks does not appear to match that extrapolated from experiment. For a more elaborate analysis of both the experimental and the computational aspects of the spectroscopy study for these two by-aryls the reader should refer to Ref. [45].

## 6. Conclusions

Nonlinear chiral spectroscopies are extremely promising techniques, which are likely to see in the coming years a powerful increase in interest for their potential application in several fields of science and technology. In this work we have reviewed the status of two-photon absorption circular dichroism, TPACD, a novel spectroscopic tool which, having been predicted theoretically now thirty five years ago, can be considered still in its infancy, having been revived only in the last few years through a series of computational and experimental breakthroughs. The sensitivity of the double L-scan technique, described in detail above, allows to fully measure the TPACD spectra of a chiral molecule in solution. In addition, one can also perform measurements of another interesting optical property in optically active molecules, *i.e.*, the two-photon absorption circular-linear polarization (TPA-CLD) [83,84]. The development of computational tools which can exploit the flexibility of modern electronic structure computer codes permits, on the other hand, the reliable prediction and simulation of nonlinear optical spectra, thus allowing for an instructive comparison of theoretical and experimental results.

We hope that this account of the experimental, theoretical and computational aspects of TPACD might further contribute to the establishment of nonlinear chiral spectroscopies in everyday routine.
